# Simvastatin does not alleviate muscle pathology in a mouse model of Duchenne muscular dystrophy

**DOI:** 10.1186/s13395-021-00276-3

**Published:** 2021-09-03

**Authors:** Olga Mucha, Paulina Podkalicka, Katarzyna Kaziród, Emilia Samborowska, Józef Dulak, Agnieszka Łoboda

**Affiliations:** 1 Department of Medical Biotechnology, Faculty of Biochemistry, Biophysics and Biotechnology, Jagiellonian University, Gronostajowa 7, 30-387 Kraków, Poland; 2 Mass Spectrometry Lab, Institute of Biochemistry and Biophysics, Polish Academy of Sciences, Warszawa, Poland

**Keywords:** 3-Hydroxy-3-methylglutaryl coenzyme A reductase inhibitors, Simvastatin, DMD, Duchenne muscular dystrophy, Angiogenesis, *mdx*

## Abstract

**Background:**

Duchenne muscular dystrophy (DMD) is an incurable disease, caused by the mutations in the *DMD* gene, encoding dystrophin, an actin-binding cytoskeletal protein. Lack of functional dystrophin results in muscle weakness, degeneration, and as an outcome cardiac and respiratory failure. As there is still no cure for affected individuals, the pharmacological compounds with the potential to treat or at least attenuate the symptoms of the disease are under constant evaluation. The pleiotropic agents, 3-hydroxy-3-methylglutaryl coenzyme A (HMG-CoA) reductase inhibitors, known as statins, have been suggested to exert beneficial effects in the mouse model of DMD. On the other hand, they were also reported to induce skeletal-muscle myopathy. Therefore, we decided to verify the hypothesis that simvastatin may be considered a potential therapeutic agent in DMD.

**Methods:**

Several methods including functional assessment of muscle function via grip strength measurement, treadmill test, and single-muscle force estimation, enzymatic assays, histological analysis of muscle damage, gene expression evaluation, and immunofluorescence staining were conducted to study simvastatin-related alterations in the *mdx* mouse model of DMD.

**Results:**

In our study, simvastatin treatment of *mdx* mice did not result in improved running performance, grip strength, or specific force of the single muscle. Creatine kinase and lactate dehydrogenase activity, markers of muscle injury, were also unaffected by simvastatin delivery in *mdx* mice. Furthermore, no significant changes in inflammation, fibrosis, and angiogenesis were noted. Despite the decreased percentage of centrally nucleated myofibers in gastrocnemius muscle after simvastatin delivery, no changes were noticed in other regeneration-related parameters. Of note, even an increased rate of necrosis was found in simvastatin-treated *mdx* mice.

**Conclusion:**

In conclusion, our study revealed that simvastatin does not ameliorate DMD pathology.

**Supplementary Information:**

The online version contains supplementary material available at 10.1186/s13395-021-00276-3.

## Background

Duchenne muscular dystrophy (DMD) is a progressive, severely debilitating, and lethal genetic disease caused by the mutations in the *DMD* gene, coding dystrophin, a 427 kDa actin-binding cytoskeletal protein, maintaining muscle fiber-extracellular matrix integrity and regulating several cellular pathways including nitric oxide (NO) production, Ca^2+^ entry, and the generation of reactive oxygen species (ROS) [1, 2]. In DMD, progressive muscle weakness, together with the loss of muscle mass and function, is a consequence of several pathological processes namely necrosis, inflammation, fibrosis, and increased oxidative stress which are results of unbalanced regenerative processes (reviewed in [3]). Recent discoveries and our research underlined also the dysregulation of angiogenesis as an additional mechanism accompanying muscle insufficiency [4–6]. As the disease progresses, patients suffering from DMD lose the ability to walk and ultimately die in the 2nd to 3rd decade of life, due to cardiac or respiratory failure [7, 8].

Taking into account the diversity of the processes which may affect DMD progression and the constant need for the development of effective therapeutics, new factors are suggested to exhibit beneficial effects on this so far incurable disease. In our previous study, we have found that lack of heme oxygenase-1 (*Hmox1*, HO-1), a heme-degrading enzyme, exerting anti-oxidant and cytoprotective activities leads to a more severe disease state through, among others, aggravation of inflammation and fibrosis [9]. *Hmox1* expression may be modulated by the plethora of compounds, including 3-hydroxy-3-methylglutaryl coenzyme A (HMG-CoA) reductase inhibitors [10], commonly known as statins, discovered 40 years ago [11] and used as lipid-lowering drugs for the treatment of hypercholesterolemia and reduction of atherosclerosis. Interestingly, in 2015, Whitehead et al. for the first time showed that simvastatin improves muscle health, reduces inflammation and oxidative stress, and increases autophagy in *mdx* animals [12] and Kim et al. reported beneficial effects on heart functions [13]. Similarly, Amor et al. suggested simvastatin treatment as a potential therapeutic agent in DMD due to its role in the regulation of cholesterol metabolism [14]. However, other studies performed with simvastatin [15] and rosuvastatin [16] did not confirm such favorable properties in animal models of DMD.

What is relevant, even the devastating role of statins on muscles has been reported, however, concentrations required to induce deleterious effects in vitro are far beyond the physiological range, being typically greater than 1 μM. Such concentrations are considerably (100–1000 times) higher than those found in vivo in humans [17].

There are also discrepant data about the incidence of different kinds of myopathy in humans after statin therapy. Previous studies indicated a high risk of such adverse effects, e.g., showing that >10% of statin users in the general population can be affected [18, 19]. Noteworthy, a recent systematic reviews of clinical trials found an extremely low risk of adverse muscle symptoms in statin takers compared with placebo controls [20, 21]. Particularly, high risk of myopathy was observed in patients who take 80 mg of simvastatin daily in comparison to those taking 20 to 40 mg, indicating exposure-dependent myotoxicity. Additionally, risk and severity may increase in the specific genetic variants, especially those affecting blood statin levels (e.g., SLCO1B1 encoding the organic anion-transporting polypeptide OATP1B1, which has been shown to regulate the hepatic uptake of statins) or in the presence of co-medications known to influence statin metabolism (e.g., cyclosporine) [22]. Statin-related muscle symptoms also appear to be exacerbated by other factors, including exercise [23], older age, and female sex [24]. On the other hand, Iwere and Hewitt showed, that even in aged patients (65+ years), the risk of statin-induced myopathy was comparable to placebo patients [25], which was also confirmed recently by Zhou et al. [26]. These data implicate that the fear of statin-caused myopathy might be in many cases overestimated. Notably, the abovementioned risk factors for statin-induced myopathy are not relevant to boys with DMD. However, it cannot be excluded that damaged muscles in DMD patients may be per se a potential risk factor for statin-associated myopathy.

Based on the published, contradictory results in the field of statin-induced muscle alterations [18–26] and their ambiguous role in muscular dystrophy [12–16] as well as our previous expertise in terms of the role of statins, including the angiogenesis process [27, 28], we aimed at the evaluation of the effect of simvastatin in *mdx* animals. We have found that simvastatin does not regulate important processes contributing to dystrophy progressions like fibrosis, inflammation, regeneration, angiogenesis, and finally, does not improve muscle functionality. Therefore, based on our results, we may conclude that simvastatin does not exert beneficial effects in the *mdx* model of DMD.

## Materials and methods

### Cell culture

C2C12 murine myoblast cells were maintained in DMEM high glucose (4.5 g/l) medium supplemented with 10% fetal bovine serum (FBS) and antibiotics: streptomycin (100 μg/ml) and penicillin (100 U/ml) (Lonza). The cells were kept at standard conditions (37°C, 5% CO_2_, 95% humidity). C2C12 myoblasts were stimulated for 24 h with 0.1 and 1 μM concentrations of simvastatin (Sigma-Aldrich).

### Animals

Animal experiments were conducted in accordance with the national and European legislation, after approval by the 2nd Institutional Animal Care and Use Committee (IACUC) in Kraków, Poland (approvals numbers: 323/2018, 301/2019, 79/2021, and 170/2021). All mice used in the study were 6-week-old WT and *mdx* male littermates or age-matched mice from generations F2 to F5, bred on a mixed C57BL/10ScSn and C57BL/6×FVB background as described by us previously [9], and housed in specific pathogen-free (SPF) conditions with water and food available ad libitum. Genotyping of animals was performed using PCR on the DNA isolated from the tails. Statin administration was continued for 1 month until the age of 10 weeks.

### Simvastatin treatment

An activation procedure was based on the published protocol [29]. Briefly, 4 mg of simvastatin (Sigma-Aldrich) was dissolved in 200 μl of ethanol. Then 300 μl of 0.1 N NaOH was added to the solution and subsequently incubated at 50°C for 2 h. The pH was brought to 7.2 by HCl, and the concentration of the stock solution was adjusted to 2 mg/ml. The stock solution was kept at 4°C. For 1 month *mdx* mice received either 10 mg/kg body weight (BW) simvastatin/day via oral gavage or solvent (vehicle group). WT animals receiving vehicle were used as a reference group. The administered dose was chosen based on the literature data [12].

### Simvastatin level estimation and pharmacokinetics

Analyzes of simvastatin and its acid form were performed at the Mass Spectrometry Laboratory at the Institute of Biochemistry and Biophysics Polish Academy of Science using the liquid chromatography-tandem mass spectrometry method (LC-MS/MS). Pharmacokinetics was assessed after 30 min, 1 h, 2 h, and 4 h after administration of simvastatin to *mdx* mice. What is more, the level of simvastatin and its metabolite was measured in the plasma and muscles at the end of 1-month-lasting experiment, 24 h after the last treatment. Muscle homogenates were prepared in 10% ethanol (1 mg muscle/4 μl homogenate mixture). Simvastatin, simvastatin acid form, and internal standard were isolated from a biological sample by liquid-liquid extraction (LLE). Biological samples (plasma, muscle homogenate) and calibrators were spiked with lovastatin (250 ng/ml in acetonitrile) (Sigma-Aldrich). Analytes were extracted with methyl tert-butyl ether (J.T. Baker) and 50 mM ammonium acetate (J.T. Baker). Finally, samples were reconstituted in 50% acetonitrile (J.T. Baker) and analyzed using the Waters Acquity Ultra Performance Liquid Chromatograph (Waters) coupled with Waters TQ-S triple-quadrupole mass spectrometer (Waters). For the instrument control and data acquisition, Waters MassLynx software was used whereas data processing was done with Waters TargetLynx (Waters). Chromatographic separation was performed using a Waters C18 column (1.7 μm, 2.1 mm × 50mm) (Waters). Mobile phase A was 2-mM ammonium acetate (J.T. Baker) with 0.1% formic acid (v/v) (J.T. Baker) in water, and mobile phase B was acetonitrile (J.T. Baker). The mass spectrometer was operated in multiple-reaction monitoring (MRM)-positive electrospray ionization (ESI+) mode. The concentrations of analytes were calculated using calibration standard mix derived from a series of calibrator samples by spiking standard stock solutions into water. Calibration curves were generated by comparing a ratio of the peak area of the analyzed compound to the peak of the internal standard against known analyte concentrations. The limits of quantification were 0.1 ng/ml and 0.5 ng/ml for simvastatin and acid form, respectively.

### Grip strength assay

Forelimb grip strength was assessed by the investigator blind to the mice’s genotype on day 26 of simvastatin administration, using a grip strength meter with a triangular pull bar (Ugo Basile) as described earlier [30, 31]. The measurements were repeated 5 times with a 1-min break in between. Following the instruction described in the TREAT-NMD SOP (https://treat-nmd.org/wp-content/uploads/2016/08/MDX-DMD_M.2.2.001.pdf), results were calculated as an average from 3 highest measurements and expressed as absolute values (*N*), or normalized to body weight (*N*/kg BW).

### Treadmill test

The treadmill exhaustion test was performed on day 29, using the Exer-3/6 (Columbus Instruments) at 15° downhill by the investigator blind to the mice’s genotype. We employed the protocol described previously [9] with modification. Briefly, after 3 daily acclimation sessions of 15 min at 8 m/min, 10-week-old male mice were subjected to an exhaustion treadmill test. Mice were warmed up at 5 m/min for 5 min, and then, they ran on the treadmill at 5 m/min for 2 min, 7 m/min for 2 min, 8 m/min for 2 min, 10 m/min for 5 min, and 12 m/min for 15 min. Afterward, the speed was increased by 1 m/min to a final speed of 20 m/min. The endpoint for each mouse was defined by the inability of the animal to remain on the treadmill after 10 stimulations with gentle touching.

### Muscle contractile properties

The specific maximum force of the tibialis anterior muscle was assessed on day 30 (1 day after the last dose) using Aurora 1300A: 3-in-1 Whole Animal System (Aurora Scientific) by measuring in situ muscle contraction in response to nerve stimulation with the trains of stimuli with increasing frequencies from 50 to 150 Hz. The determined maximal force was further normalized to the muscle weight. In fatigue protocol, we evaluated the decrease in the force of a muscle over time due to continuous stimulation of 50 Hz for 30 s. For analysis, we determined the time after which a drop in muscle force by 50% of basal value was obtained. Analysis was performed with the researcher blind to the genotype of the mice.

### Blood cell count

The blood was collected directly from the *vena cava* to the EDTA-coated tubes and the total number of white blood cells (WBC) and the percentage of granulocytes, monocytes, and lymphocytes among WBC was analyzed using scil Vet abc (Horiba ABX).

### Histological and immunofluorescent analysis of the muscles

For histological assessment muscles were collected and pre-treated with OCT medium (Leica) for few minutes directly after collection. Afterward, they were transferred to new, OCT-containing tubes, frozen in liquid nitrogen-cooled isopentane, and stored at −80°C. Then, 10-μm-thick sections were cut on a cryotome (Leica), placed on the previously coated with poly-l-lysine slides, air-dried for at least 2 h, and kept at −20°C for further analyses. Hematoxylin and eosin (H&E) staining and Masson’s trichrome were performed on the 4% buffered formalin-fixed (pH 7.4) frozen sections. For **H&E**, tissue sections were incubated in Mayer’s hematoxylin (Sigma-Aldrich) for 12 min, rinsed with tap water (15 min), and stained for 1.5 min in 0.1% eosin solution (96% EtOH and distilled water, 7:3) (Sigma-Aldrich). After the staining, the sections were incubated in increasing concentrations (70%, 96% (×2), 99.8% (×2)) of aqueous EtOH (POCH), then 2 times in xylene (Sigma-Aldrich) and sealed in Histofluid medium (Chemilab). **Masson’s trichrome** (Trichrome Stain (Masson) Kit, Sigma-Aldrich) was performed following the manufacturer’s protocol. After the staining, the sections were incubated in increasing concentrations (70%, 96% (×2), 99.8% (×2)) of aqueous EtOH, then 2 times in xylene and sealed in Histofluid medium (Chemilab). Analyses were conducted according to our previous studies [4, 9, 32] after taking pictures of the whole tissues. The assessment of inflammation and fibrosis extent was conducted using arbitrary units, respectively: 0—no signs of inflammation/collagen deposition; 1—any sign of leukocyte infiltration and myofiber swelling/collagen deposition; 2—visible inflammation, myofiber swelling, and rhabdomyolysis/collagen deposition; 3—signs of inflammation, myofiber swelling, and rhabdomyolysis which take around half of a field of view/collagen deposition takes up around half of the field of view; and 4—a substantial part of the muscle in the field of view is infiltrated and degenerated/collagen deposition takes the substantial part of the field of view. The analysis of centrally nucleated fibers (CNF) indicating the level of regeneration was performed based on H&E staining; 10–15 pictures/tissue section were randomly taken and the percentage of CNF among all fibers was calculated.

Immunofluorescent staining of **CD31/α-SMA-**positive vessels was performed as described by us previously with slight modifications [33]. Primary antibodies: rat anti-mouse CD31 (BD Pharmingen, 550274) and rabbit anti-human α-SMA (Abcam, ab5694) were used followed by the incubation with secondary antibodies: goat anti-rat Alexa Fluor 488 (for detection of α-SMA) and goat anti-rabbit Alexa Fluor 568 (for detection of CD31). Pictures of the whole tissue were taken, and CD31/α-SMA-positive vessels were analyzed quantitatively per muscle area. The results were presented as a number of vessels per area. **Pax7/laminin** co-staining was performed as described before [5, 31]. **Necrosis** was assessed by the immunofluorescent staining of the IgG/IgM/IgA (goat anti-mouse IgG, IgM, IgA Alexa Fluor 488 antibody, Thermo Fisher Scientific) with laminin α2 (rabbit anti-mouse antibody, Abcam, ab11576; secondary antibody: goat anti-rabbit Alexa Fluor 568) and showed as a percentage of necrotic fibers in the stained muscle. Evaluation of the **muscle cross-sectional area (CSA) and the mean fiber area** was determined by semi-automatic muscle analysis using segmentation of histology (SMASH) [34] based on immunofluorescent staining of laminin.

The stainings were visualized under Nikon Eclipse T*i* fluorescent microscope. All histological assessments were analyzed by the investigator blind to the mice group using ImageJ software. If necessary, the brightness and/or contrast were adjusted to all of the pictures equally.

### Determination of serum CK and LDH concentrations

To estimate the activity of CK and LDH diagnostic Liquick Cor-CK and Liquick Cor-LDH kits were used, respectively, according to the manufacturer protocols (Cormay). The blood was collected from *vena cava* and was allowed to clot at room temperature for 30 min and then centrifuged at 4°C for 10 min at 2000*g*. The assay was performed using a clear serum, without the signs of hemolysis. The absorbance values were then converted to CK and LDH (U/l).

### RNA isolation, reverse transcription (RT), and quantitative real-time PCR (qRT-PCR)

The collected muscles were protected in RNA*later* (Sigma-Aldrich), snap-frozen in liquid nitrogen, and stored at −80°C for downstream analyses. RNA was isolated as in our previous study [9] using the Chomczynski-Sacchi method [35]. Its quality and concentration were determined by the NanoDrop Spectrophotometer (Thermo Fisher Scientific). qRT-PCR was performed as described previously [9] using StepOne Plus Real-Time PCR (Applied Biosystems - Thermo Fisher Scientific) and SYBR Green PCR Master Mix (Sigma-Aldrich), specific primers (listed in Table 1), and cDNA obtained in the RT reaction with recombinant M-MuLV reverse transcriptase (Thermo Fisher Scientific). *Eef2* was used as a housekeeping gene. The expression of miR-1, miR-133a-3p, and miR-206 was normalized to the constitutive SNORD68 gene (LNA miRCURY RT-PCR Kit and miRCURY LNA^TM^ miRNA PCR Assay). Relative quantification of gene expression was calculated based on the comparative cycle threshold (C_t_) method (according to the 2^-ΔCt^ formula where ΔC_t_ = C_t gene of interest_ – C_t *Eef2/SNORD68*_) and presented as the relative expression in comparison to vehicle-treated WT animals.

**Table 1:** The sequences of primers used for the determination of gene expression by qRT-PCR

Gene	*Sequence 5’-3’*
**Ang 1**	*F: CAGTGGCTGCAAAAACTTGA* *R: TGGGCCATCTCCGACTTCAT*
**Col1a1**	*F: CGATCCAGTACTCTCCGCTCTTCC* *R: ACTACCGGGCCGATGATGCTAACG*
**Cxcl12**	*F: CCTTCAGATTGTTGCACGGCT* *R: CCCACCACTGCCCTTGCATC*
**Eef2**	*F: AGAACATATTATTGCTGGCG* *R: AACAGGGTCAGATTTCTTG*
**Hmox1**	*F: CCTCACTGGCAGGAAATCATC* *R: CCTCGTGGAGACGCTTTACATA*
**Kdr**	*F: CGGCCAAGTGATTGAGGCAG* *R: ATGAGGGCTCGATGCTCGCT*
**Mmp11**	*F: CAGATTTGGTTCTTCCAAGG* *R: AGATCTTGTTCTTCTCAGGAC*
**Myh3**	*F: TCTAGCCGGATGGTGGTCC* *R: GAATTGTCAGGAGCCACGAA*
**Spp1**	*F: CCATCTCAGAAGCAGAATCTCCTT* *R: GGTCATGGCTTTCATTGGAATT*
**Tgfb1**	*F: GGATACCAACTATTGCTTGAG* *R: TGTCCAGGCTCCAAATATAG*
**Vegfa**	*F: ATGCGGATCAAACCTCACCAA* *R: TTAACTCAAGCTGCCTCGCCT*

### Enzyme-linked immunosorbent assay (ELISA)

The fragments of the muscles were snap-frozen in liquid nitrogen, homogenized in 1% Triton X-100 in PBS using TissueLyser (QIAGEN), and centrifuged (7000*g*, 10 min, 4°C). The protein lysates were collected, and the total protein concentration was measured by bicinchoninic acid (BCA, Sigma-Aldrich) assay. One hundred micrograms of protein lysate was used to determine the level of vascular endothelial growth factor (VEGF), fibroblast growth factor-2 (FGF2), endoglin (CD105), and stromal cell-derived factor-1 (SDF-1) according to the vendor’s instructions (R&D Systems). To assess the level of osteopontin (OPN), 750 times-diluted mouse serum was subjected to the test and the concentration was quantified based on the absorbance values according to the manufacturer’s protocol (R&D Systems).

### Statistical analyses

Data are presented as mean ± SEM. Differences between groups were tested for statistical significance using the one-way ANOVA followed by Tukey’s post hoc test; *p* < 0.05 was considered significant. The outliers were identified based on Grubb’s test.

## Results

### Simvastatin treatment does not influence CK and LDH activities in *mdx *mice

**Fig. 1:**
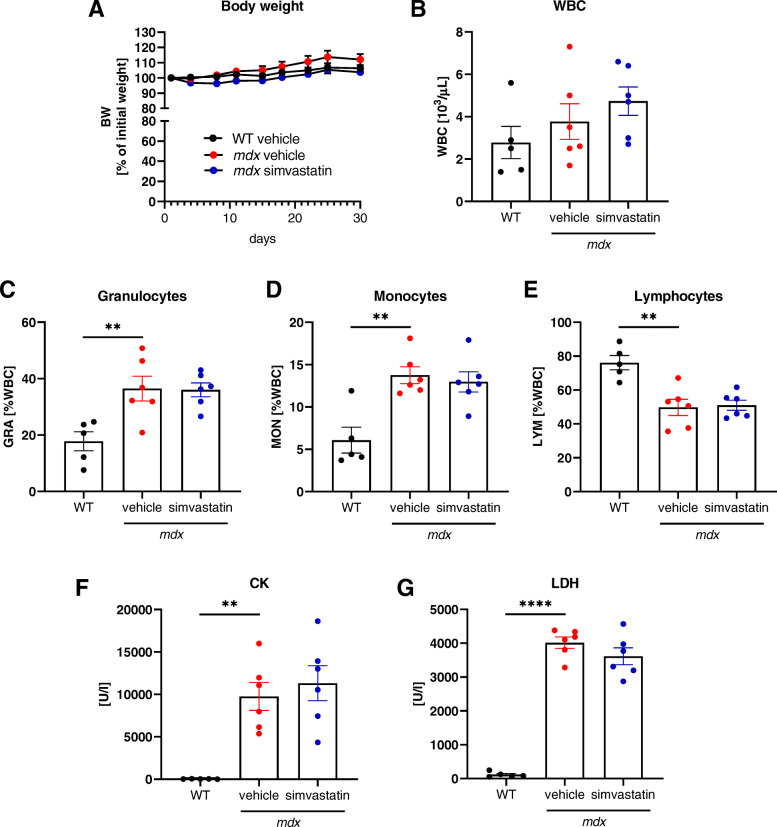
Simvastatin treatment does not affect WBC abundance and CK and LDH activity. **A** Changes in the body weight (BW) over the time of simvastatin treatment; *n*=10–15/group. Results presented as a percentage of initial weight (mean ± SEM). **B** Unchanged number of white blood cells (WBC) in the peripheral blood; *n*=5–6/group. Increased percentage of **C** granulocytes and **D** monocytes in dystrophic animals compared to the WT mice without the effect of simvastatin. **E** Diminished percentage of lymphocytes in dystrophic animals compared to the WT mice without simvastatin-related changes. **F** Augmented serum activity of CK in *mdx* animals with no effect of the drug in simvastatin-treated dystrophic mice; *n*=5–6/group. **G** Increased serum LDH activity in *mdx* mice without simvastatin-related alterations; *n*=5–6/group. Results shown as a mean ± SEM; ^**^p < 0.01 and ^****^p < 0.0001

Administration of simvastatin for 1 month in a dose of 10 mg/kg BW/day by oral gavage did not result in the change in BW of the animals (Fig. 1A). Moreover, the blood cell analysis did not reveal any detrimental effect of statin treatment with regard to WBC (Fig. 1B), granulocytes (Fig. 1C), monocytes (Fig. 1D), and lymphocytes (Fig. 1E), measured at the end of the experiment.

One of the hallmarks of DMD is the elevated level of serum markers of muscle damage [36]. Accordingly, the activity of CK (Fig. 1F) and LDH (Fig. 1G) was potently increased in the dystrophic animals, with no effect upon drug administration.

### Simvastatin treatment fails to improve the exercise capacity, forelimb grip strength, and contractile properties of the *mdx *mice

**Fig. 2:**
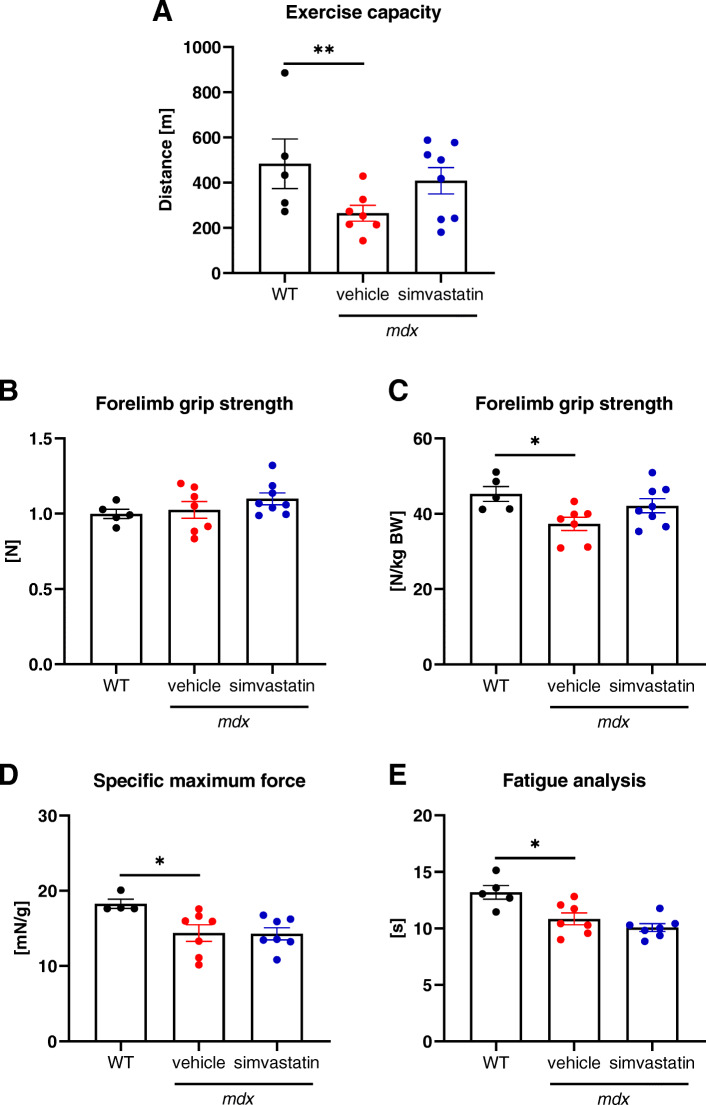
The exercise capacity, grip strength, and muscle contractility are not altered upon simvastatin treatment. **A** Downhill running treadmill test presented as the total distance run by mice during the test *n*=5–8/group, mean ± SEM. **B** Forelimb grip strength analysis is shown as the absolute values or **C** normalized to body weight (*N*/kg BW); *n*=5–8/group, mean ± SEM. **D** Specific maximum force and **E** fatigue analysis of the tibialis anterior muscle; in situ muscle contractile measurements using the Aurora system; *n*=4–7; mean ± SEM; ^*^p < 0.05, ^**^p < 0.01

To assess the functional effect of statin treatment, we carried out three types of tests. The treadmill exhaustion experiment did not show any difference in the running capacity of simvastatin-treated *mdx* mice when compared to the vehicle-treated mice (Fig. 2A). Similarly, muscle function in dystrophic animals after simvastatin administration was not altered in the forelimb grip strength test (Fig. 2B, C). Furthermore, when tibialis anterior contractility was measured using the Aurora system, no apparent improvement was noticed upon treatment, both with regard to the maximal force measurement and in fatigue analysis (Fig. 2D, E), respectively.

### Simvastatin treatment does not affect inflammation in the dystrophic muscles

**Fig. 3:**
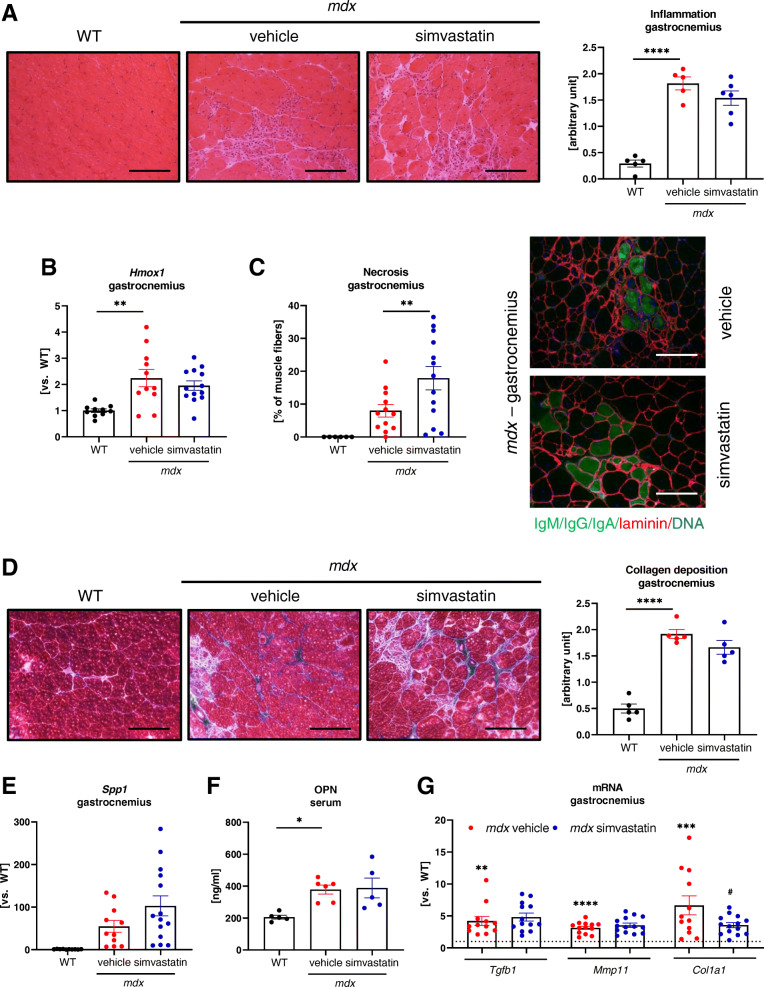
Simvastatin treatment does not attenuate inflammation and fibrosis in gastrocnemius of *mdx* mice. **A** Representative pictures of hematoxylin and eosin (H&E) staining of the gastrocnemius muscle with semi-quantitative analysis of inflammation; scale bar: 100 μm; mean ± SEM; *n*=5–6/group. **B** Unaffected by simvastatin treatment expression of *Hmox1* gene in gastrocnemius, presented as a mean ± SEM; *n*=10–13; qRT-PCR. **C** Necrosis assessment using immunofluorescent staining of IgM/IgG/IgA binding (green) with laminin (red) and its calculation presented as a mean ± SEM; *n*=6–13/group; scale bar: 100 μm. **D** Representative photos of Masson’s trichrome staining with semi-quantitative analysis of collagen deposition showing no changes in the extent of fibrosis in gastrocnemius of simvastatin-treated animals; scale bar: 100 μm; *n*=5/group. **E** Unaffected by simvastatin treatment expression of *Spp1* gene in gastrocnemius, presented as a mean ± SEM; *n*=10–14; qRT-PCR. **F** The protein level of serum marker of fibrosis, OPN, *n*=10–13/group, mean ± SEM; ELISA. **G** Unaltered by the treatment expression of fibrotic markers: *Tgfb1* and *Mmp11*, in gastrocnemius of *mdx* mice and a significant decrease in *Col1a1* mRNA; *n*=12–14/group, WT level marked with the dotted line; qRT-PCR. Data are presented as mean ± SEM; ^*^for *mdx* vehicle vs. WT and # for *mdx* simvastatin vs. *mdx* vehicle comparison; ^*^p < 0.05, ^**^p < 0.01, ^***^p < 0.001, ^****^p < 0.0001, and ^#^*p* < 0.05

A dramatic exacerbation in inflammatory cell infiltration was observed both in the gastrocnemius as well as in the diaphragm of dystrophic animals; however, H&E staining did not reveal any effect of simvastatin on inflammation, regardless of the type of the analyzed muscle (Fig. 3A, Supplementary Fig. 1A). Accordingly, heme oxygenase-1 (*Hmox1*), a marker of inflammation and oxidative stress augmented in dystrophic animals, was affected by the treatment neither in gastrocnemius (Fig. 3B) nor the diaphragm (Supplementary Fig. 1B). The muscle necrosis, as assessed by the immunofluorescent staining of the IgG/IgM/IgA, the membrane-impermeable markers, was even intensified upon simvastatin administration, as evidenced by a higher number of necrotic fibers in the gastrocnemius muscle of simvastatin-treated *mdx* mice (Fig. 3C).

### Simvastatin does not reduce fibrosis in dystrophic animals

In dystrophic muscles, collagen deposition was clearly visible; however, the simvastatin treatment did not attenuate fibrosis as shown by semi-quantitative analysis of trichrome staining both in gastrocnemius (Fig. 3D) and in diaphragm (Supplementary Fig. 1C) muscles. Of note, the mRNA (*Spp1*) and protein level of OPN, one of the markers of fibrosis, elevated in *mdx* mice, were not affected by statin treatment (Fig. 3E, F, Supplementary Fig. 1D). Furthermore, the augmented expression of other fibrotic factors, including transforming growth factor-beta 1 (*Tgfb1*) and matrix metalloproteinase 11 (*Mmp11*) was unchanged by simvastatin in both analyzed muscles (Fig. 3G, Supplementary Fig. 1E). Although there was a significant decrease in collagen type I alpha 1 chain (*Col1a1)* in the gastrocnemius (Fig. 3G), no such effect was found in the diaphragm (Supplementary Fig. 1E).

### Simvastatin treatment does not influence muscle regeneration

**Fig. 4:**
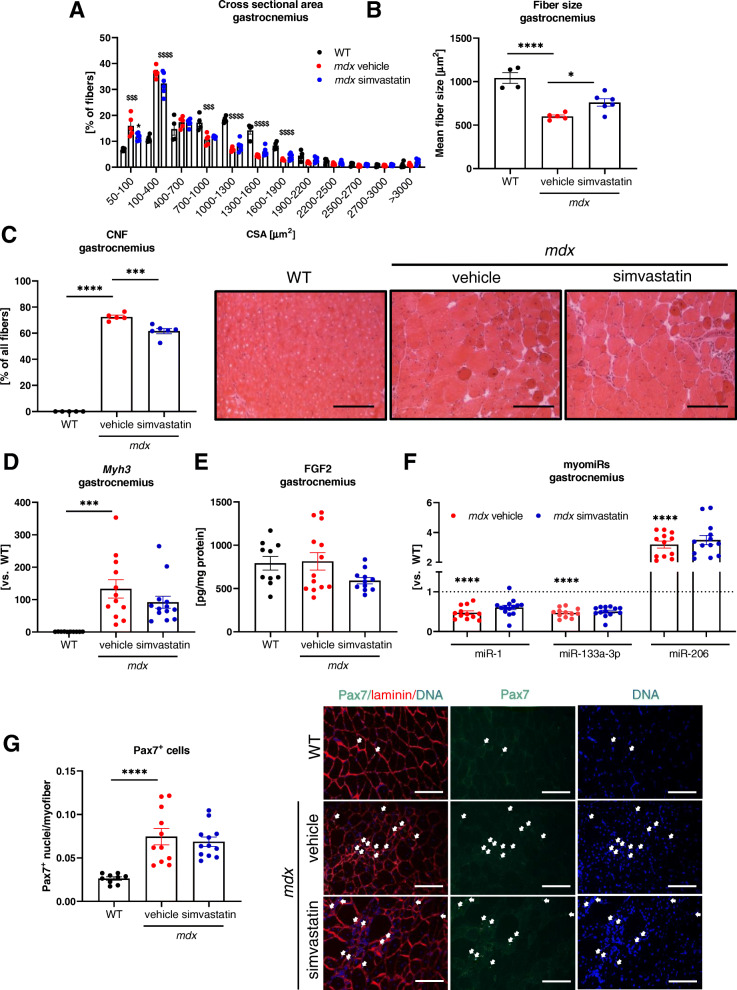
Simvastatin treatment does not influence muscle regeneration in gastrocnemius muscle of *mdx* mice. **A**, **B** Quantification of muscle fiber size based on laminin staining (not shown); *n*=5–6/group; mean ± SEM. **C** The quantitative analysis and representative photos of centrally nucleated fibers (CNF) performed based on H&E staining showing a drop in CNF number in *mdx* animals treated with simvastatin when compared to the vehicle group; scale bar: 100 μm; *n*=5–6/group; mean ± SEM. **D** Expression of myosin heavy chain isoform-coding gene: *Myh3*, presented as mean ± SEM; *n*=10–13; qRT-PCR. **E** The protein level of FGF2 in the gastrocnemius muscle of dystrophic animals upon simvastatin treatment; *n*=10–13/group; presented as mean ± SEM; ELISA. **F** myomiRs: miR-1, miR-133a-3p, and miR-206 expression upon simvastatin treatment in gastrocnemius muscle of *mdx* animals presented as mean ± SEM; *n*=12–14; WT level marked with the dotted line; LNA qRT-PCR. **G** The quantitative analysis and representative photos of Pax7-positive satellite cells in the gastrocnemius muscle; scale bar: 100 μm; *n*=9–12/group; mean ± SEM; ^*^for *mdx* simvastatin vs. *mdx* vehicle and ^$^for *mdx* vehicle vs. WT comparison; ^*^p < 0.05, ^***^p < 0.001, ^****^p < 0.0001, ^$$$^*p* < 0.001, ^$$$$^*p* < 0.0001

The size of the fibers appeared to be smaller in vehicle-treated *mdx* mice when compared to the WT counterparts (Fig. 4A, B), whereas the mean fiber size was increased in the gastrocnemius muscle of dystrophic animals upon simvastatin treatment (Fig. 4A, B). At the same time, the percentage of CNF was lower when simvastatin-treated *mdx* mice were compared to the vehicle group; however, still, it was greatly increased in comparison to WT animals, in which almost no fibers with central nuclei have been found (Fig. 4C). On the contrary, no changes in fiber size, mean fiber size, and CNF were noticed after statin treatment in the diaphragm, although the differences between WT and *mdx* animals were still prominent (Supplementary Fig. 1F-H).

Importantly, also the expression of embryonic myosin heavy chain isoform *Myh3*, encoding eMyHC especially relevant in the matter of muscle regeneration [37], despite being increased in the dystrophin-lacking vehicle group, was unaffected by statin treatment (Fig. 4D). In addition, the protein level of FGF2, upregulated during regeneration [38], was not significantly changed in the gastrocnemius of simvastatin-receiving animals (Fig. 4E). As microRNAs, especially the so-called myomiRs, play an important role in muscle regeneration [39], we decided to check the expression of the three miRNAs: miR-1, miR-133a-3p, and miR-206. A significant downregulation of miR-1 and miR-133-3p, and upregulation of miR-206 were evident in vehicle-treated *mdx* animals, but simvastatin was not able to change their expression (Fig. 4F). Also, when the number of Pax7-positive satellite cells was estimated in the gastrocnemius muscles, we observed their prominent elevation in dystrophic animals treated with the vehicle with no further effect of simvastatin administration (Fig. 4G).

### Simvastatin treatment does not affect angiogenic markers and vascularization in dystrophic muscles

**Fig. 5:**
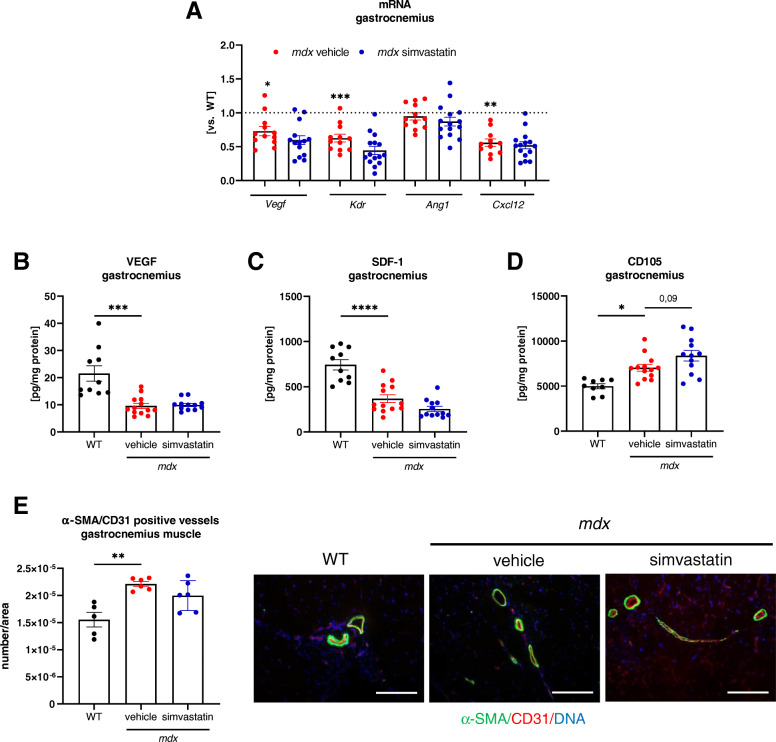
Simvastatin treatment does not influence the angiogenic markers in the gastrocnemius muscle in dystrophic animals. **A** Decreased mRNA level of angiogenesis-related genes *Vegfa*, *Kdr*, and *Cxcl12* in the gastrocnemius muscle of vehicle-treated *mdx* mice and no changes in *Ang1*; *n*=11–15/group; presented as mean ± SEM; WT level marked with the dotted line; qRT-PCR. The unaffected protein level of **B** VEGF, **C** SDF-1, and **D** CD105 in the gastrocnemius muscle of statin-receiving animals; *n*=9–13/group; presented as mean ± SEM, ELISA. **E** The quantitative analysis and representative photos of the blood vessels performed based on CD31/α-SMA double staining showing no difference in **the** gastrocnemius muscle of *mdx* animals treated with simvastatin when compared to the vehicle group; *n*=5–6/group; presented as mean ± SEM; scale bar: 100 μm; ^*^p < 0.05, ^**^ *p* < 0.01, ^***^p < 0.001, ^****^p < 0.0001

Recent discoveries underline the role of dysregulation of angiogenesis in DMD pathology [4, 5, 31, 40]. In our previous studies, we have found the concentration- and cell-type dependent effect of statins on VEGF synthesis and overall angiogenic activity [27, 41, 42]. However, in the C2C12 mouse, myoblast cell line simvastatin at the physiologically relevant concentrations (0.1–1 μM) did not affect *Vegfa* (Supplementary Fig. 3). In vivo, simvastatin treatment of dystrophic animals did not cause any alterations in the expression of already decreased in most cases angiogenic genes, such as *Vegfa*, kinase insert domain receptor (*Kdr*), angiopoietin-1 (*Ang1*), and C–X–C motif chemokine 12 (*Cxcl12*), also known as gene coding stromal cell-derived factor 1 (SDF-1) (Fig. 5A). Importantly, VEGF (Fig. 5B), as well as SDF-1 (Fig. 5C) and endoglin (CD105) (Fig. 5D) protein levels, were also unaffected. Furthermore, no effect of statin treatment on the analyzed factors was observed in the diaphragm, both on mRNA and protein level (Supplementary Fig. 2A-D). Additionally, when the number of CD31^+^/α-SMA^+^ vessels was evaluated in gastrocnemius muscle, no differences were noted after statin delivery (Fig. 5E). Interestingly, a significant rise in the number of CD31^+^/α-SMA^+^ vessels was observed in the diaphragm, showing differences between various muscles (Supplementary Fig. 2E).

### The level of simvastatin and its active form declines rapidly after administration

**Fig. 6:**
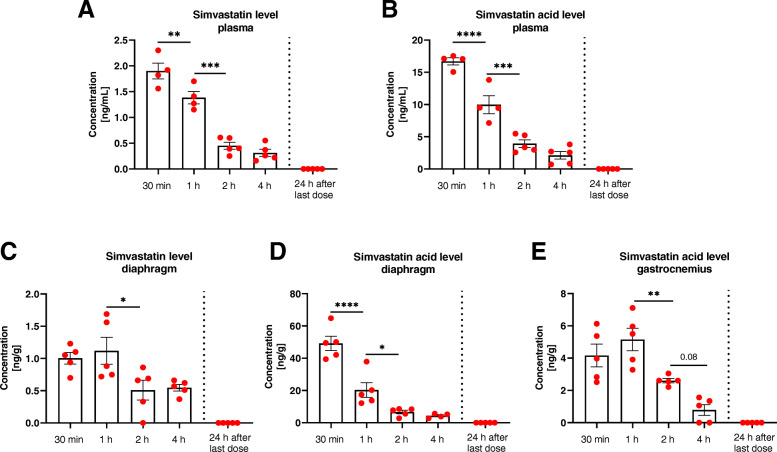
Simvastatin and simvastatin active metabolite concentration declines rapidly after administration. Simvastatin level measured in **A** plasma and **C** diaphragm. Simvastatin acid analyzed in **B** plasma**, D** diaphragm, and **E** gastrocnemius after 30 min, 1 h, 2 h, and 4 h, and 24 h after the last dose of the drug administration; *n*=4–5; ^*^p < 0.05, ^**^p < 0.01, ^***^p < 0.001, and ^****^p < 0.0001

In order to determine, whether the lack of simvastatin effect in our studies could be explained by low plasma levels and insufficient muscle distribution of the drug, we performed the pharmacokinetic analysis of the simvastatin and its active, acid form. Our results showed a detectable concentration of both simvastatin and its metabolite in plasma (Fig. 6A, B) and in the diaphragm (Fig. 6C, D) in all-time points (30 min–4 h). Interestingly, in gastrocnemius muscle, we were not able to detect simvastatin, whereas the concentration of the active metabolite was considerably lower than in the diaphragm (Fig. 6E). Importantly, the level of active form of simvastatin in the plasma and diaphragm was considerably higher than the corresponding level of simvastatin, what was expected as the activation of simvastatin was performed before in vivo administration via oral gavage.

Nevertheless, in all tested samples, the level of both forms declines rapidly over time. In addition, when simvastatin and simvastatin acid form concentrations were assessed 24 h after the last dose of monthly administration, their level was undetectable in all samples.

## Discussion

Despite many years of intensive and profound studies, DMD remains an incurable disease. The newest, most promising therapies, using the latest advances in genetic modification, namely CRISPR/Cas9 technology, are still far from clinical introduction and acceptance. Thus, glucocorticoids constantly serve as a gold standard therapy for patients suffering from DMD. Unfortunately, except for undoubtful beneficial impact on DMD pathology, their daily administration was shown to exert many side effects leading to, among others, osteoporosis, diabetes, or muscle atrophy [43]. As there is a constant need to investigate novel strategies, which could at least attenuate the severity of the disease, many researchers focus not only on new drug discoveries but also on the repurposing of the already existing ones.

HMG-CoA reductase inhibitors, commonly known as statins, seem to be the perfect choice for such investigation. Despite the still ongoing discussion regarding statin-induced myopathy, myositis, and rhabdomyolysis [17, 19, 23, 44] recent studies describe that the benefits of the treatment, outweigh the possible risks which, of note, are usually not relevant to DMD boys [21, 26]. Several studies demonstrated the positive effects of statins on overall skeletal muscle health, including their anti-inflammatory and anti-fibrotic properties [45, 46]. Whitehead et al. already showed in 2015 the protective influence of simvastatin in dystrophic animals [12], and similarly, promising results were published this year by Amor et al. [14]. On the other hand, the 2020 publication by Verhaart et al. described the lack of the effect of such treatment [15]. Also, when different statins were investigated by other groups, the results were inconclusive. Pravastatin, another FDA-approved cholesterol-lowering drug, was proposed to upregulate the expression of utrophin A [47], an autosomal homolog of dystrophin, compensating for its loss in DMD muscles [48]. On the other hand, Finkler et al. demonstrated no beneficial effects of rosuvastatin and even a visible accretion of inflammation was remarked upon treatment [16].

In our study, simvastatin was not able to alleviate dystrophic muscle pathology and even a significantly higher percentage of necrotic fibers was detected in dystrophic animals after drug delivery, which might suggest deterioration of the muscles’ condition. Nevertheless, no other tested parameters seem to confirm aggravation of the disease as no elevation in CK level, strongly associated with statin-induced myopathy [44], was observed after 1 month of simvastatin administration. Moreover, no significant or alarming systemic changes were observed in regards to the total WBC or distinguished subpopulation, e.g., granulocytes, lymphocytes, and monocytes, when simvastatin-treated animals were compared to the *mdx* vehicle group at the end of the experiment.

In contrast to the results obtained by Whitehead et al., which showed visibly reduced inflammatory cell infiltration [12], we did not observe the anti-inflammatory potential of simvastatin. Moreover, the expression of the *Hmox1* gene, coding anti-oxidant, and cytoprotective HO-1 enzyme was also not affected by the treatment. Similar results were obtained by Verhaart et al. [15], who reported no effect on inflammation even with the prolonged by 2 months, in comparison to us, time of drug administration. Furthermore, in opposition to the published data suggesting the anti-fibrotic role of simvastatin in *mdx* animals [12, 13], we did not observe any effect of the treatment on fibrosis, neither in histological assessment of collagen deposition nor expression of fibrosis-related genes. Those observations were confirmed by the evaluation of OPN expression, a recently described biomarker of DMD associated with regeneration, inflammation, and fibrosis [49, 50], which was also not changed by simvastatin.

When gastrocnemius muscle was investigated, we noticed a significant increase in the mean myofiber size and a decline in the number of CNF. However, no alterations were noticed in other regeneration-related parameters, such as the expression of *Myh3* gene, FGF2 protein, muscle-specific myomiRs (miR-1, miR-133a-3p, and miR-206), or the number of Pax7-positive satellite cells. Similarly, no effect of simvastatin on dystrophic muscle regeneration was demonstrated by Whitehead et al. and Verhaart et al. [12, 15].

More importantly, in our study, simvastatin treatment influenced neither the exercise capacity and forelimb grip strength of the animals nor the muscle contractility properties. Interestingly, when a measurement of the specific force of the muscle was performed by Whitehead et al. [12] and Verhaart et al. [15], they showed a significant improvement and lack of any effect, respectively.

To expand the already described knowledge, we decided to investigate the modulation of angiogenesis, another aspect of DMD progression. The improvement of endothelial function and vasculoprotective action are well-recognized statin effects [28, 51]. Importantly, our previous studies revealed angiogenesis alterations in *mdx* mice [4, 5, 31] which might be also age-dependent [40]. Similarly, in the present work, a significant drop in the expression of angiogenic genes in the gastrocnemius muscle was found on the mRNA and protein level but without any impact of simvastatin treatment. Correspondingly, no changes in the abundance of CD31/α-SMA double-positive blood vessels were observed. Despite the elevated number of the vessels in the diaphragm, no other investigated factors were affected, suggesting no profound effect on angiogenesis as the result of simvastatin administration. Noteworthy, it shows that simvastatin might not be influencing various muscles in the same manner.

In summary, our study, in accordance with work by Verhaart et al. [15] and Finkler et al. [16], did not support the hypothesis about the positive effect of statins in DMD. The discrepant findings found by, e.g., Whitehead et al. [12] might be related to several divergences in the applied methodology, including age and genetic background of the mice, type and dose of the statin that was used, route of administration, and length of time the drug was given to the animals. Nevertheless, diverse strategies give an undoubtful chance to investigate the effects of statins from different perspectives and various stages of disease progression. In our study, the dose of simvastatin was 10 mg/kg/d. In that matter, the applied approach was similar to the one used by other groups [12, 15]. Moreover, despite the most promising results obtained by Whitehead et al. when simvastatin was provided in food and water [12], we strongly believe that oral gavage administration is more relevant, taking into account a short half-life of simvastatin in the circulation. What is more, this route of delivery gives the opportunity to more precisely control the given dose. Importantly, our pharmacokinetic experiment showed the detectable level of the active metabolite, simvastatin acid, in the plasma and tested muscles, with the highest concentration obtained in the diaphragm. Noteworthy, due to rapid decline in the concentration over time, we cannot exclude the possibility that a higher amount of the drug is required for the beneficial outcome of the treatment, and as measured by us, the level is much lower than reported by Whitehead et al. [12], where administration of simvastatin at the same dose in the food resulted in plasma levels of 170 ng/mL. On the other hand, our data are more comparable to Verhaart et al. results [15], although it is not clearly stated whether the level of statin or its metabolite was measured in that study. The lack of protective effect of simvastatin may be related to the relatively short duration of the treatment. However, we even observed a significant increase in the percentage of necrotic fibers after a month-long experiment that does not fully support conducting longer administration of simvastatin. Of note, an extension to 12 weeks in Verhaart et al. study did not increase the plasma simvastatin level and resulted in no improvement [15]. Furthermore, it has to be emphasized that mice used by Whitehead et al. and Verhaart et al. were maintained on a C57BL/6J genetic background [12, 15], whereas in our study, we utilized mice bred on C57BL/10ScSn and C57BL/6×FVB mixed background. This was due to our previous and ongoing studies regarding various modulators of DMD progression, originally initiated by investigation of the role of HO-1 in dystrophic mice by crossing HO-1-deficient animals (C57BL/6×FVB background) with *mdx* mice (C57BL/10ScSn background) [9]. Importantly, our results are in line with Verhaart et al. study, indicating that the lack of simvastatin effect is background-independent.

Nonetheless, discrepancies in the performed experiments should be carefully considered in future studies.

## Conclusion

In conclusion, we demonstrated that simvastatin does not significantly influence DMD pathology in *mdx* mice. Our results do not support the hypothesis that statins are a potential therapeutic option in DMD.

## Supplementary Information

**Additional file 1:** Supplementary Figure 1. **Simvastatin treatment does not change inflammation and fibrosis in the diaphragm of** ***mdx*** **mice. (A)** Representative pictures of hematoxylin and eosin (H&E) staining with semi-quantitative analysis of inflammation; scale bar: 100 μm; mean ± SEM; n=4-6/group. **(B)** Unaffected by simvastatin treatment expression of *Hmox1* gene, presented as a mean ± SEM; n=10-13, qRT-PCR. **(C)** Representative photos of Masson’s trichrome staining with semi-quantitative analysis of collagen deposition showing no alterations in the extent of fibrosis of simvastatin-treated animals; scale bar: 100 μm; n=5-6/group. **(D)** Unaffected by simvastatin treatment expression of *Spp1* gene, presented as a mean ± SEM; *n*=10-13; qRT-PCR **(E)**. Unchanged by the treatment expression of fibrotic markers: *Tgfb1, Mmp11,* and *Col1a1* mRNA; n=12-13/group, WT level marked with the dotted line; qRT-PCR. Data are presented as mean ± SEM. No changes in diaphragm **(F)** cross-sectional area (CSA) **(G)**, muscle fiber size, and **(H)** percentage of CNF were observed in statin-treated *mdx* mice; *n*=5-6; presented as mean ± SEM; * for *mdx* simvastatin vs. *mdx* vehicle and $ for *mdx* vehicle vs. WT comparison; *p < 0.05, ** *p* < 0.01, ***p < 0.001, ****p < 0.0001, ^$^ *p* < 0.05, ^$$$^ *p* < 0.001, ^$$$$^ *p* < 0.0001. Supplementary Figure 2. **Simvastatin** **treatment** **has** **no** **impact** **on** **angiogenic** **markers** **in** **the** **diaphragm** **muscle** **of** ***mdx*** **mice**. **(A)** Decreased mRNA level of angiogenesis-related *Vegfa* in diaphragm muscle of vehicle-treated *mdx* mice and no changes in *Kdr, Ang1,* and *Cxcl12*; n=12-13/group; presented as mean ± SEM; WT level marked with the dotted line; qRT-PCR. The unaffected protein level of **(B)** VEGF, **(C)** SDF-1, and **(D)** CD105 in diaphragm muscle of statin-receiving animals; n=5-6/group; presented as mean ± SEM, ELISA. **(E)** The quantitative analysis and representative photos of blood vessels performed based on CD31/α-SMA double staining showing a significant exacerbation in diaphragm muscle of *mdx* animals treated with simvastatin when compared to the vehicle group; n=5-6/group; presented as mean ± SEM; scale bar: 100 μm; * *p* < 0.05, ***p < 0.001, ****p < 0.0001. Supplementary Figure 3. **Simvastatin does not affect** ***Vegfa*** **mRNA in murine myoblasts.** Expression of *Vegfa* in C2C12 cell line after simvastatin treatment, *n*=6/group, presented as mean ± SEM.

## Data Availability

The datasets used and/or analyzed during the current study are available from the corresponding author on reasonable request.

## Abbreviations

α-SMA: Alpha-smooth muscle actin
*Ang1*: Angiopoietin 1
CD105: Cluster of differentiation 105, Endoglin
CD31: Cluster of differentiation 31
CK: Creatine kinase
*Col1a1*: Collagen type I alpha 1
CNF: Centrally nucleated fibers
CSA: Cross-sectional area
*Cxcl12*, SDF-1: C–X–C motif chemokine ligand 12, stromal cell-derived factor 1
DGC: Dystrophin-glycoprotein complex
DMD: Duchenne muscular dystrophy
eNOS: Endothelial nitric oxide synthase
FGF2: Fibroblast growth factor-2
HMEC-1: Human microvascular endothelial cells
HMG-CoA: 3-Hydroxy-3-methylglutaryl coenzyme A
*Hmox1,* HO-1: Heme oxygenase-1
HUVEC: Human umbilical vein endothelial cells
*Kdr*: Receptor for VEGF (VEGF-R2)
LDH: Lactate dehydrogenase
*Mmp11*: Matrix metalloproteinase 11
mSCs: Muscle satellite cells
*Myh3*, eMyHC: Myosin heavy chain 3, embryonic myosin heavy chain
Pax7: Paired Box 7
ROS: Reactive oxygen species
*Spp1*, OPN: Secreted phosphoprotein 1, osteopontin
*Tgfb1*: Transforming growth factor beta 1
*Vegfa*, VEGF: Vascular endothelial growth factor A
