# Supplementary figures and images for: Simvastatin does not alleviate muscle pathology in a mouse model of Duchenne muscular dystrophy
Source: Skelet Muscle. 2021 Sep 3;11:21. doi: 10.1186/s13395-021-00276-3 (PMC8414747; doi:10.1186/s13395-021-00276-3)

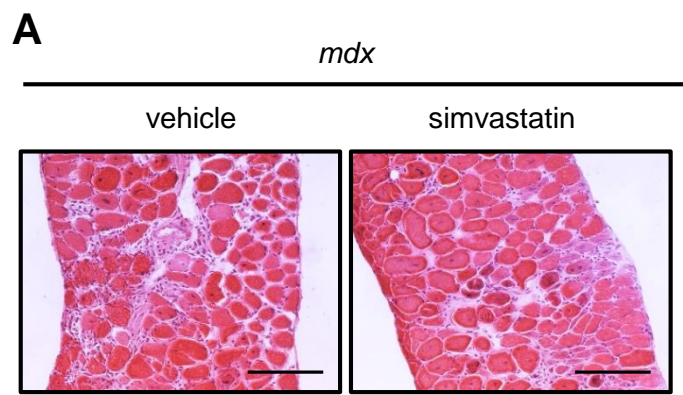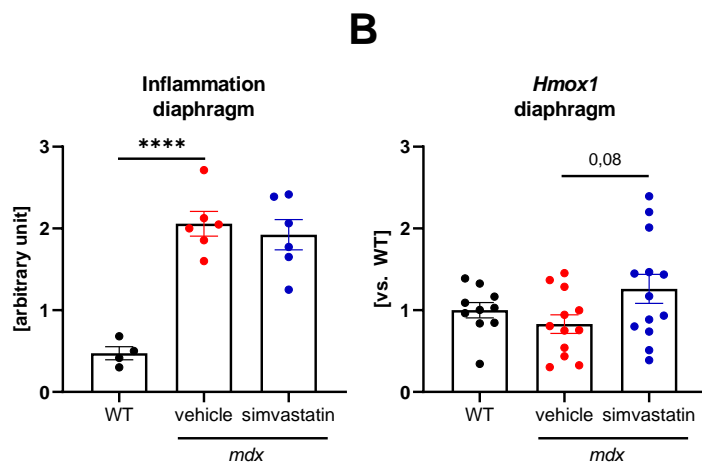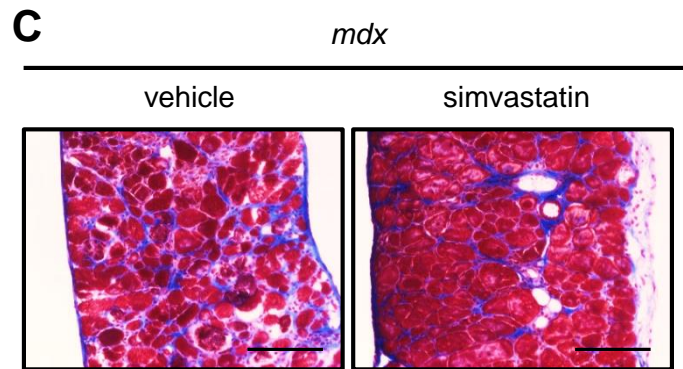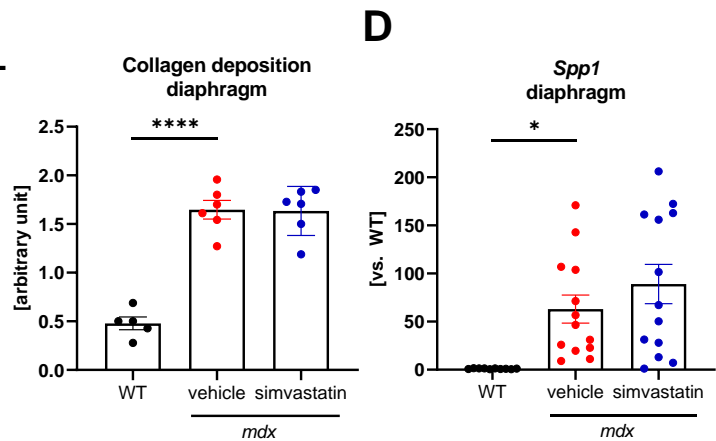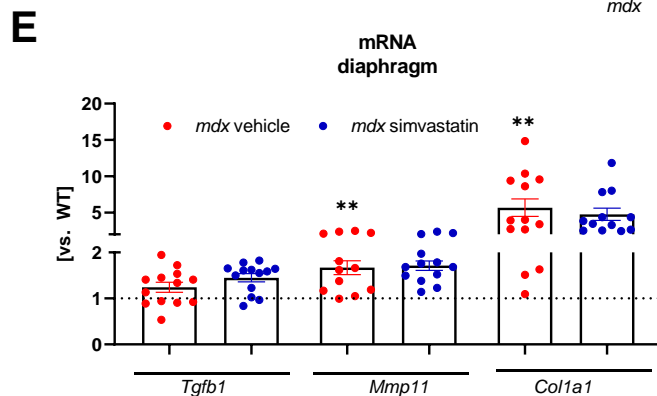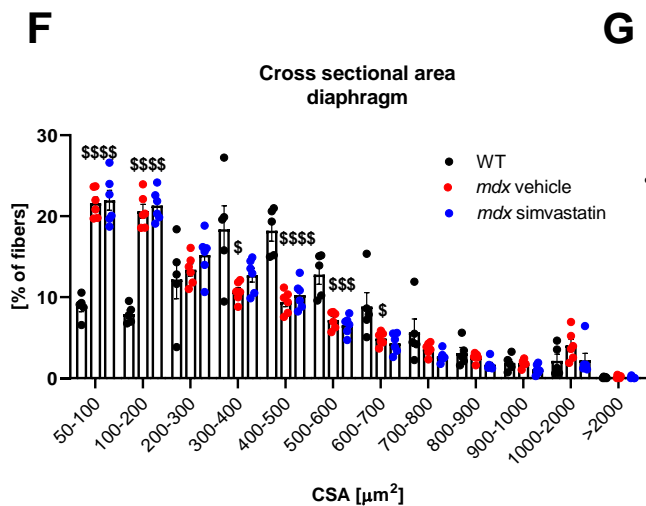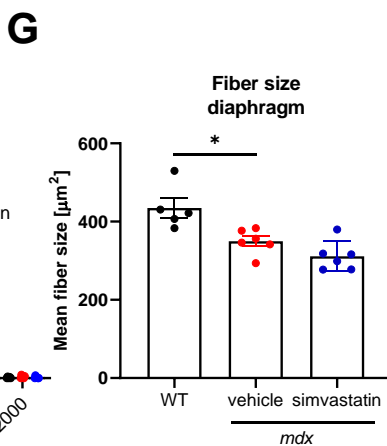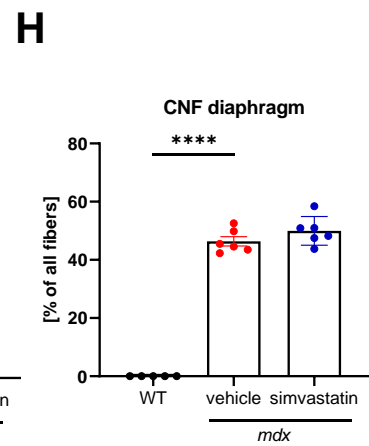

Supplementary Fig. 1

**A**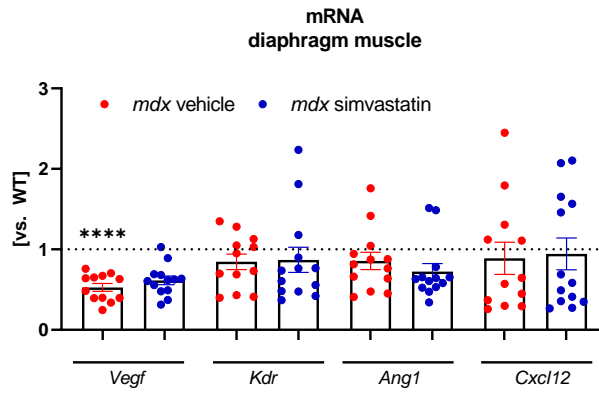**B**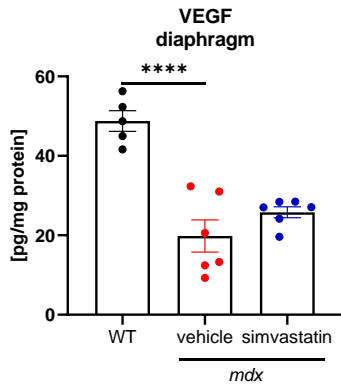**C**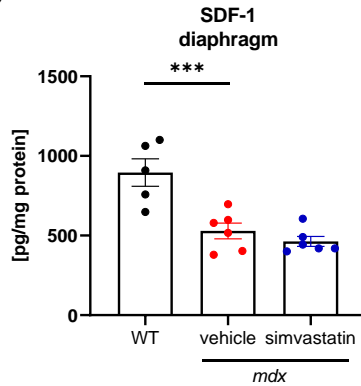**D**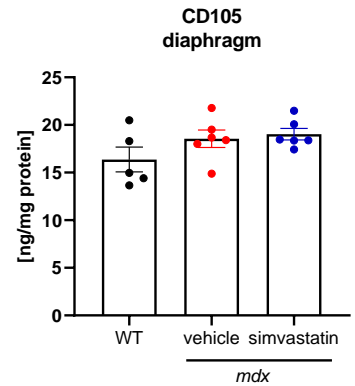**E**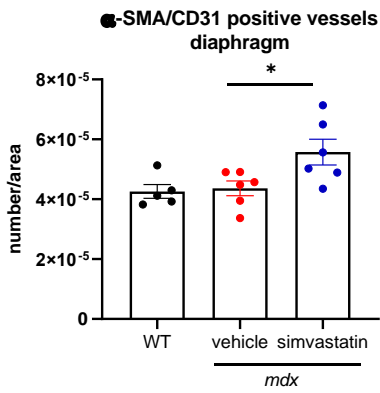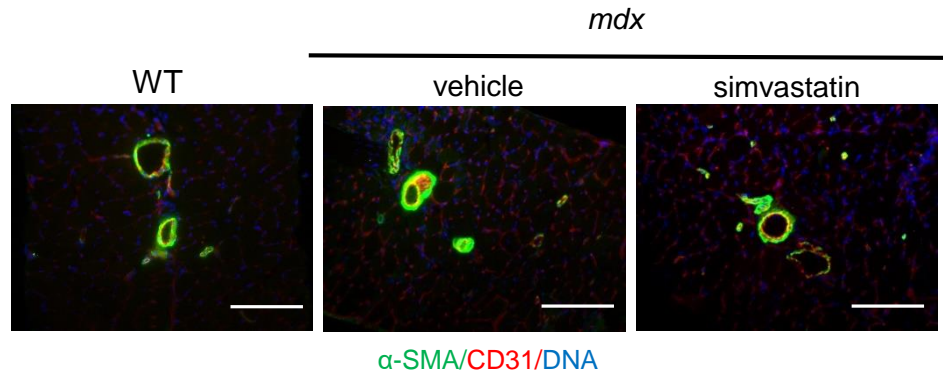

***Vegfa* expression in C2C12**

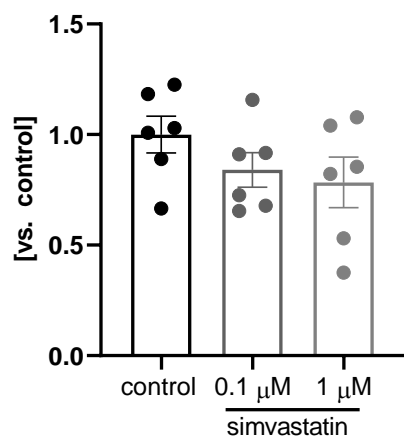

Supplement: Supplementary file 1 — Additional file 1: Supplementary Figure 1. Simvastatin treatment does not change inflammation and fibrosis in the diaphragm of mdx mice. (A) Representative pictures of hematoxylin and eosin (H&E) staining with semi-quantitative analysis of inflammation; scale bar: 100 μm; mean ± SEM; n=4-6/group. (B) Unaffected by simvastatin treatment expression of Hmox1 gene, presented as a mean ± SEM; n=10-13, qRT-PCR. (C) Representative photos of Masson’s trichrome staining with semi-quantitative analysis of collagen deposition showing no alterations in the extent of fibrosis of simvastatin-treated animals; scale bar: 100 μm; n=5-6/group. (D) Unaffected by simvastatin treatment expression of Spp1 gene, presented as a mean ± SEM; n=10-13; qRT-PCR (E). Unchanged by the treatment expression of fibrotic markers: Tgfb1, Mmp11, and Col1a1 mRNA; n=12-13/group, WT level marked with the dotted line; qRT-PCR. Data are presented as mean ± SEM. No changes in diaphragm (F) cross-sectional area (CSA) (G), muscle fiber size, and (H) percentage of CNF were observed in statin-treated mdx mice; n=5-6; presented as mean ± SEM; * for mdx simvastatin vs. mdx vehicle and $ for mdx vehicle vs. WT comparison; *p < 0.05, ** p < 0.01, ***p < 0.001, ****p < 0.0001, $ p < 0.05, $$$ p < 0.001, $$$$ p < 0.0001. Supplementary Figure 2. Simvastatin treatment has no impact on angiogenic markers in the diaphragm muscle of mdx mice. (A) Decreased mRNA level of angiogenesis-related Vegfa in diaphragm muscle of vehicle-treated mdx mice and no changes in Kdr, Ang1, and Cxcl12; n=12-13/group; presented as mean ± SEM; WT level marked with the dotted line; qRT-PCR. The unaffected protein level of (B) VEGF, (C) SDF-1, and (D) CD105 in diaphragm muscle of statin-receiving animals; n=5-6/group; presented as mean ± SEM, ELISA. (E) The quantitative analysis and representative photos of blood vessels performed based on CD31/α-SMA double staining showing a significant exacerbation in diaphragm muscle of mdx animals treated wi [file 13395_2021_276_MOESM1_ESM.pdf]
